# Downregulation of miR-221-3p and upregulation of its target gene PARP1 are prognostic biomarkers for triple negative breast cancer patients and associated with poor prognosis

**DOI:** 10.18632/oncotarget.21561

**Published:** 2017-10-06

**Authors:** Ling Deng, Qianqian Lei, Yu Wang, Zhu Wang, Guiqin Xie, Xiaorong Zhong, Yanping Wang, Nianyong Chen, Yan Qiu, Tianjie Pu, Hong Bu, Hong Zheng

**Affiliations:** ^1^ Laboratory of Molecular Diagnosis of Cancer, Clinical Research Center for Breast, State Key Laboratory of Biotherapy, National Collaborative Innovation Center for Biotherapy, West China Hospital, Sichuan University, Chengdu, China; ^2^ Department of Radiation Oncology, Chongqing Cancer Institute & Hospital & Cancer Center, Chongqing, China; ^3^ Cancer center, West China Hospital, Sichuan University, Chengdu, China; ^4^ Department of Pathology, West China Hospital, Sichuan University, Chengdu, China

**Keywords:** triple negative breast cancer, prognosis, microRNA, miR-221-3p, PARP1

## Abstract

The purpose of this study was to identify microRNAs (miRNAs) closely associated with the prognosis of triple-negative breast cancer (TNBC) and their possible targets. This study recruited 125 early-stage TNBC patients, including 40 cases in the experimental group (20 cases with poor prognoses vs. 20 cases with good prognoses) and 85 cases in the validation group (27 cases with poor prognoses vs. 58 cases with good prognoses). In the experimental group, miRNA microarray showed 34 differentially expressed miRNAs in patients with different prognoses. We selected 5 miRNAs for validation. The differential expression of miR-221-3p was further verified in the experimental and validation groups using real-time polymerase chain reaction (PCR). High miR-221-3p expression was associated with better 5-year disease-free survival (DFS) (HR = 0.480; 95% CI, 0.263–0.879; *p* = 0.017) of TNBC patients. High expression of its target gene *PARP1* predicted poorer 5-year DFS (HR = 2.236, 95% CI, 1.209-4.136, *p* = 0.010). MiR-221-3p down-regulated PARP1 by targeting its 3'-untranslated region.

In conclusion, low miR-221-3p expression may contribute to the poor outcome of TNBC patients through regulating *PARP1*. MiR-221-3p likely plays a role as a PARP1 inhibitor by directly regulating PARP1 expression, thereby affecting the prognoses of TNBC patients.

## INTRODUCTION

Triple-negative breast cancer (TNBC), which accounts for 10-20% of breast cancer patients, is negative for estrogen receptor (ER), progesterone receptor (PR), and human epidermal growth factor receptor 2 (HER2) [1]. Due to lack of specific molecular markers and therapeutic targets, chemotherapy is currently the major method of adjuvant therapy for early-stage TNBC patients and for the treatment of recurrent TNBC patients. However, the long-term treatment effect of chemotherapy is unsatisfactory, resulting in poor prognosis [2]. Compared with other types of breast cancer, TNBC is more aggressive and prone to early recurrence and metastasis [3]. Investigation of the prognostic markers of TNBC is important to distinguish patients with different prognoses and help to select the appropriate patients and guide treatment.

MicroRNAs (miRNAs) are a class of endogenous non-coding small RNAs found in eukaryotes that have regulatory functions [4]. In cancers, miRNAs may act as tumor suppressors or promoters [5] and are associated with tumor diagnosis [6], molecular typing [6], prognostic judgment [7], and treatment [8]. Radojicic *et al* found that the expression levels of miR-221, miR-21, miR-210, miR-10b, miR-145, miR-205, and miR-122a were significantly different between cancer and normal tissues of TNBC patients [9]. Aberrant expression is associated with breast cancer metastasis [10]. Meanwhile, miRNA expression in tumors may serve as a prognostic indicator. A miRNA expression profile composed of 6 miRNAs can determine the prognosis of stage I non-small cell lung cancer [11]. In breast cancer, a blood-based four-miRNA signature (miR-18b, miR-103, miR-107, and miR-652) can predict tumor recurrence and overall survival (OS) in TNBC patients [7]. Thus, miRNA expression has a certain value for the prognostic judgment of patients with tumor.

Drugs based on DNA repair mechanisms, such as poly (ADP-Ribose) polymerase 1 (PARP1) inhibitors, Chk inhibitors, and ATM/ATR inhibitors, play an important role in anti-tumor therapy. PARP1 is a DNA repair protein involved in single-strand break repair and homologous recombination repair in the genome [12]. PARP1 expression is higher in tumors than in normal tissues [13], and several PARP1 inhibitors, such as olaparib, have entered Phase I-III clinical trials [14]. Despite being limited to TNBC and other tumors, the possible appropriate population for PARP1 inhibitors and their molecular markers for efficacy prediction remain unclear. It is necessary to explore new molecular markers that are associated with the efficacy and prognostic judgment of PARP1 inhibitors. PARP1 has been found to be regulated by miRNAs, such as miR-124 [15] and miR-223 [16]. However, the relationship between miRNA expression and PARP1 in TNBC is still unclear, and the relationship between miRNA expression and TNBC prognosis has not been elucidated.

In the present study, we selected TNBC patients with different prognoses as subjects. We detected the miRNA markers that may predict the prognosis of patients and then analyzed the possible target gene. The results suggested that downregulation of miR-221-3p and upregulation of its target gene *PARP1* are prognostic biomarkers for TNBC patients and associated with poor 5-year disease-free survival (DFS). MiR-221-3p likely plays a role as a PARP1 inhibitor by directly regulating *PARP1*, thereby affecting the prognosis of patients with TNBC.

## RESULTS

### Clinicopathological characteristics

All breast cancers in this study were TNBC, which are negative for ER, PR, and HER2. All of the 125 patients were female, with an average age of 51.6 years (range 25 – 81 years) and a median follow-up time of 75 months (range 1 – 171 months). The general clinical features are summarized in Table 1. A total of 94 patients received anthracyclines in their adjuvant chemotherapy. The experimental group included 20 patients with poor prognoses, who exhibited cancer recurrence or death within five years of surgery, and 20 patients with good prognoses, who showed DFS greater than five years. In the experimental group, the baseline state was similar between patients with good prognoses and those with poor prognoses. The validation group included 27 patients with poor prognoses, and 58 patients with good prognoses. In the validation group, except for the fact that patients with poor prognoses showed more lymph node metastasis, the other clinicopathological features exhibited no differences between patients with different prognoses.

**Table 1:** General clinical features of patients

Item	All cases (n = 125)	Experimental group (40 cases)			Validation group (85 cases)		
		Good-prognosis (20 cases)	Poor-prognosis (20 cases)	P^*^	Good-prognosis (58 cases)	Poor-prognosis (27 cases)	P^*^
**Age**							
Mean	51.6	52.6	54.7	0.576	50.2	51.7	0.573
Range	25-81	36-75	38-79		27-77	25-81	
**Menopausal status**							
Premenopausal	60 (48%)	8 (40%)	8 (40%)	1.00	32 (55.2%)	12 (44.4%)	0.357
Postmenopausal	65 (52%)	12 (60%)	12 (60%)		26 (44.8%)	15 (55.6%)	
**T stage**							
1	19 (15.2%)	3 (15%)	3 (15%)	0.971	8 (13.8%)	5 (18.5%)	0.481
2	88 (70.4%)	12 (60%)	13 (65%)		45 (77.6%)	18 (66.7%)	
3	13 (10.4%)	3 (15%)	2 (10%)		4 (6.9%)	4 (14.8%)	
4	5 (4%)	2 (10%)	2 (10%)		1 (1.7%)	0	
**Lymph node metastasis**							
No	55 (44%)	9 (45%)	6 (30%)	0.327	33 (56.9%)	7 (25.9%)	***0.008***
Yes	70 (56%)	11 (55%)	14 (70%)		25 (43.1%)	20 (74.1%)	
**Ki-67**							
<14%	20 (16%)	5 (25%)	3 (15%)	0.693	9 (15.5%)	3 (11.1%)	0.835
≥14%	105 (84%)	15 (75%)	17 (85%)		49 (84.5%)	24 (88.9%)	
**Adjuvant Chemotherapy**							
Anthracyclines	46 (36.8%)						
Taxanes	10 (8%)						
Anthracyclines combined with taxanes	48 (38.4%)						
Others	21 (16.8%)						

^*^χ^2^ test or Fisher’ exact test.

In addition to the 125 patients in experimental and validation groups, we also selected 12 TNBC patients with paired cancer and paracancerous normal tissues as the control group.

### MiRNA expression profile differences in cancer tissues of TNBC patients

The miRCURY^™^ LNA Array system was used to detect the miRNA expression profiles in the cancer tissues of the experimental group (20 cases with good prognoses vs. 20 cases with poor prognoses). Differentially expressed miRNAs between good and poor prognoses patients were defined as > 1.5 times the expression difference with *p* < *0.05* in a t-test. A total of 266 miRNAs were found to be differentially expressed in patients with different prognoses (these 266 miRNAs were named as list I). A total of 103 miRNAs showed lower expression levels in patients with poor prognoses, and the other 163 miRNAs showed higher expression levels in patients with poor prognoses.

We also compared the miRNA expression profiles between paired cancer and normal tissues in the control group. The miRNA expression with no difference between paired cancer and normal tissues was defined as < 1.5 times the expression difference. A total of 1,330 miRNAs expression were found with no difference (these 1,330 miRNAs were named as list II). We compared the miRNAs in list I and list II, and 232 miRNAs were found in both lists. These 232 miRNAs were excluded from list I. Thirty-four miRNAs were determined to be the differentially expressed miRNAs between TNBC patients with different prognoses. All 34 miRNAs were down-regulated in patients with poor prognoses (Supplementary Table 1).

### MiR-221-3p expressed differentially in cancer tissues of TNBC patients

**Figure 1:**
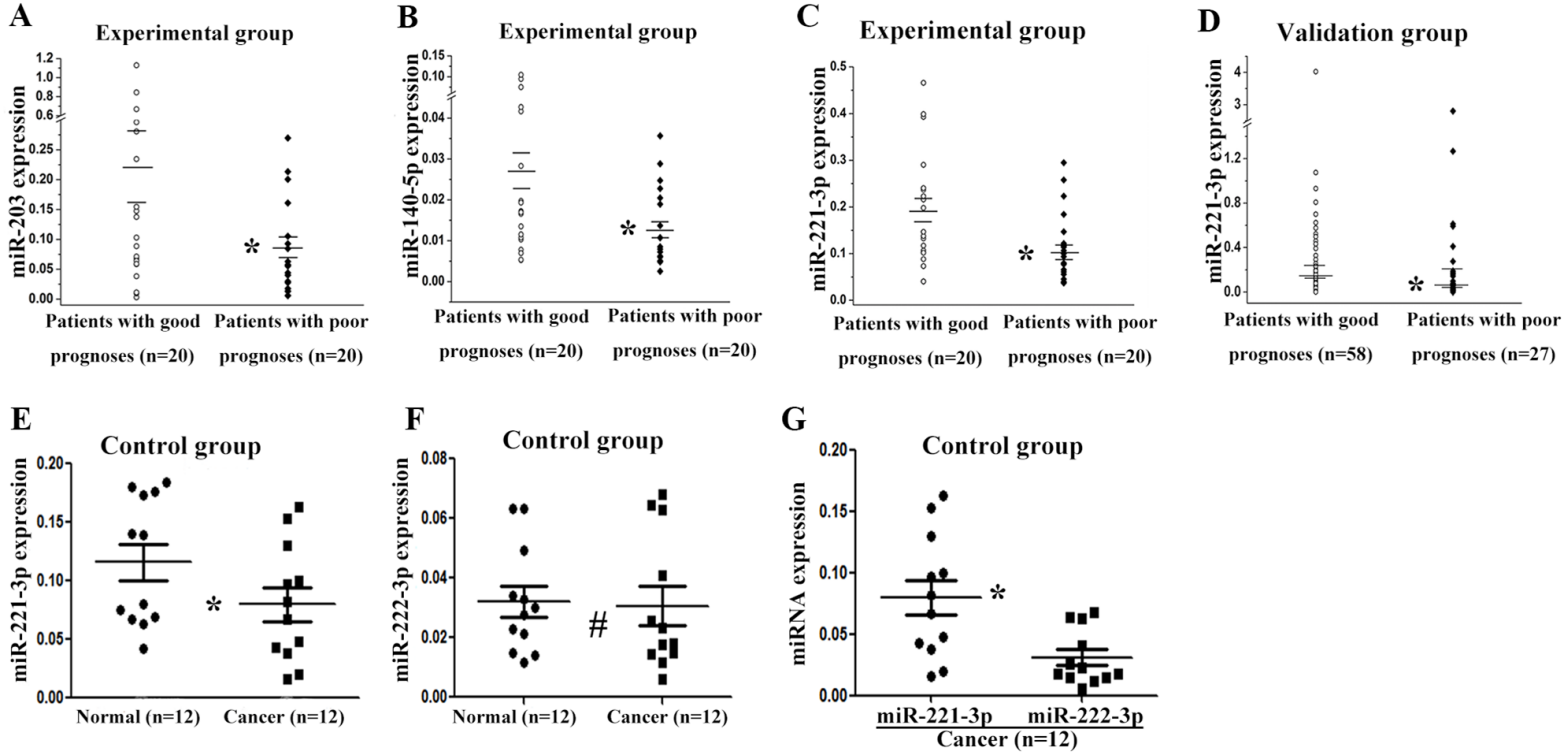
Validation of differentially expressed miRNAs by real-time PCR The relative expression levels of miR-203 **(A)**, miR-140-5p **(B)**, and miR-221-3p **(C)** are shown for 40 specimens of the experimental group. **(D)** Relative expression of miR-221-3p in the validation group. **(E)** Relative expression of miR-221-3p in 12 paired cancer and normal tissues in the control group. **(F)** Relative expression of miR-222-3p in the control group. **(G)** Relative expression of miR-221-3p and miR-222-3p in the cancer tissues of the control group. Long horizontal marks represent the mean expression of miRNAs in patients with different prognoses, and short horizontal marks represent the standard error of the mean miRNA expression. ^*^ *p* < 0.05; # *p* > 0.05; A, B, C, E, F, G, t test; D, non-parametric test.

Five miRNAs were selected for further validation using real-time PCR. MiR-34a-3p, miR-203, miR-221-3p, and miR-4532 were four of top ten down-regulated miRNAs in the patients with poor prognoses. MiR-140-5p was previously reported to possibly affect the development and progression of tumor [17]. First, we tested 5 miRNAs in the experimental group. The expression levels of miR-203, miR-221-3p, and miR-140-5p were significantly lower in patients with poor prognoses (*p* < *0.05*, *t*-test, Figure 1A – 1C). This result was consistent with the miRNA microarray data. No difference was exhibited in the expression levels of miR-34a-3p and miR-4532 (data not shown). Next, we tested miR-203, miR-221-3p, and miR-140-5p in the validation group. The results showed that the expression level of miR-221-3p in cancer tissue was significantly lower in patients with poor prognoses (*p* < *0.05*, non-parametric test, Figure 1D). There was no significant difference in the expression of miR-203 or miR-140-5p between the two groups of patients (data not shown).

In order to further determine the expression of miR-221-3p, the control group was tested. The miR-221-3p was down-regulated in cancer tissues compared with paired normal tissues, and this finding was consistent with the miRNA microarray data (*p* = 0.011, *t*-test, Figure 1E). The expression of miR-222-3p was also tested in the control group, but no difference was observed between cancer and normal tissues (*p* = 0.759, *t*-test, Figure 1F). The correlation between miR-221-3p and miR-222-3p was performed in the 12 TNBC cancer tissues, which showed no correlation between the two miRNAs, with a correlation coefficient 0.110 (p = 0.733). And miR-221-3p showed higher expression level than miR-222-3p in cancer tissues (*p* = 0.008, *t*-test, Figure 1G).

### MiR-221-3p was an independent prognostic factor for TNBC patients

**Figure 2:**
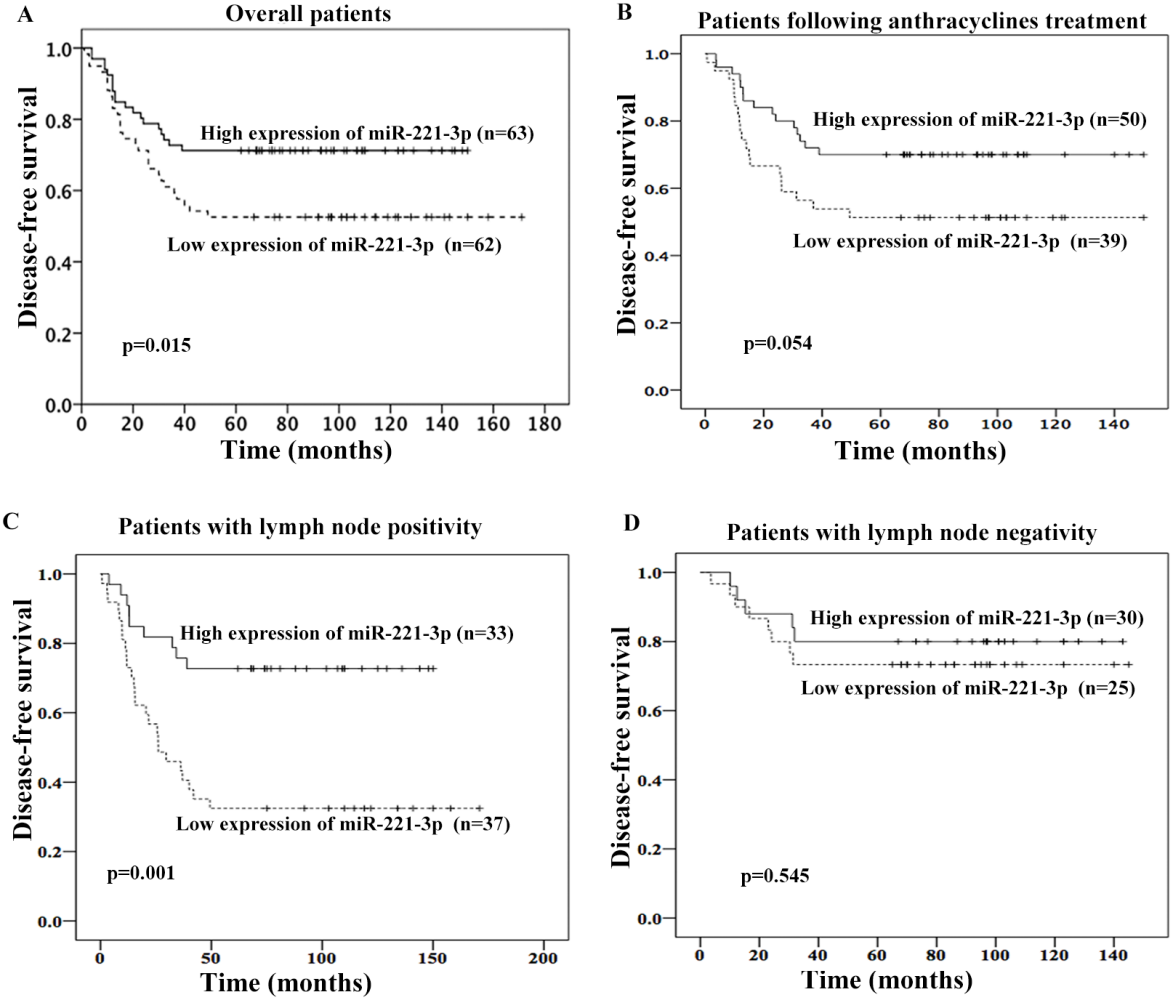
Association between miR-221-3p expression and 5-year disease-free survival (DFS) of TNBC patients High miR-221-3p expression was associated with better 5-yearDFS in overall patients **(A)**, patients following anthracyclines treatment **(B)**, or patients with lymph node positivity **(C)**. But no association was observed in patients with lymph node negativity **(D)**.

We evaluated the association between miR-221-3p expression and DFS of TNBC patients by Kaplan-Meier and Cox proportional hazard regression analyses. We combined the experimental and validation groups and then regrouped the 125 patients into two groups according to the median expression level of miR-221-3p. The baseline data of the low miR-221-3p expression and high miR-221-3p expression groups are shown in Supplementary Table 2. No correlation was observed between the miR-221-3p expression level and menopausal status, T stage, lymph node metastasis, or Ki-67 expression level (*p >* 0.05, χ^2^ test). The median DFS was 77 months in patients with high miR-221-3p expression levels and 70 months in those with low miR-221-3p expression levels. The difference was statistically significant according to the Kaplan-Meier survival analysis and log-rank test, *p* = 0.015 (Figure 2A). Univariate and multivariate analyses showed that high miR-221-3p expression (HR = 0.480; 95% CI, 0.263 – 0.879; *p* = 0.017) was an independent good prognostic factor for the 5-year DFS of TNBC patients (Table 2). In addition, lymph node metastasis was also an independent prognostic factor for TNBC patients.

**Table 2:** Univariate and multivariate analyses for 125 TNBC patients

Factor	Univariate analysis	P^*^	Multivariate analysis	P^*^
	HR (95% CI)		HR (95% CI)	
**Menopausal status (premenopausal vs. postmenopausal)**	1.271 (0.713-2.267)	0.416	1.200 (0.666-2.162)	0.545
**T stage (T3/4 vs. T1/2)**	1.256 (0.587-2.688)	0.557	1.061 (0.489-2.304)	0.880
**Lymph node metastasis (yes vs. no)**	2.421 (1.277-4.590)	**0.007**	2.340 (1.220-4.486)	**0.010**
**Ki-67 (≥14%vs. <14%)**	1.450 (0.615-3.415)	0.396	1.488 (0.624-3.545)	0.370
**MiR-221-3p (high expression vs. low expression)**	0.486 (0.268-0.882)	**0.018**	0.480 (0.263-0.879)	**0.017**

Abbreviation: HR, Hazard ratio; CI, confidence interval.

^*^ log-rank test.

In patients who received adjuvant anthracyclines chemotherapy, those with low miR-221-3p expression exhibited a trend for shorter 5-year DFS (*p* = 0.054, Figure 2B). We also evaluated the prognostic role of miR-221-3p in patients with different lymph node status. In patients with positive lymph node, patients with low miR-221-3p showed lower 5-year DFS (adjusted HR = 0.279; 95%CI, 0.128-0.610; *p*=0.001; adjusted by menopausal status, Ki67 index and T stage; Figure 2C). But this was not observed in patients with negative lymph node (Figure 2D).

### PARP1 was a potential target gene and associated with DFS

Some potentially actionable pathways were found in TNBC, such as DNA repair pathway, PI3K/mTOR pathway, RAS/RAF/MEK pathway, cell-cycle checkpoints, JAK/STAT pathway and so on [18]. Several targeted therapeutic agents are currently under clinical investigation, such as PARP1 inhibitors, PI3K inhibitors, MEK inhibitors, and inhibitors of the cancer stem-cell population [18]. These pathways contained some essential genes, such as *BRCA1/2*, *PARP*, *PIK3CA*, *AKT*, *PTEN*, *KRAS*, *BRAF*, *EGFR*, *FGFR*, *INPP4B*, *CDK6*, *RB1*, *CCND* and *JAK2*. We checked whether these essential genes were in the range of the predictive targets of miR-221-3p in the miRanda (http://www.microrna.org/microrna/home.do), miRBase (http://microrna.sanger.ac.uk/) and TargetScan (http://www.targetscan.org/) databases. We found *PARP1* and *PTEN* might be target genes of miR-221-3p. *PTEN* has been validated to be a target gene of miR-221-3p [19]. Thus, we chose *PARP1* for further validation.

**Figure 3:**
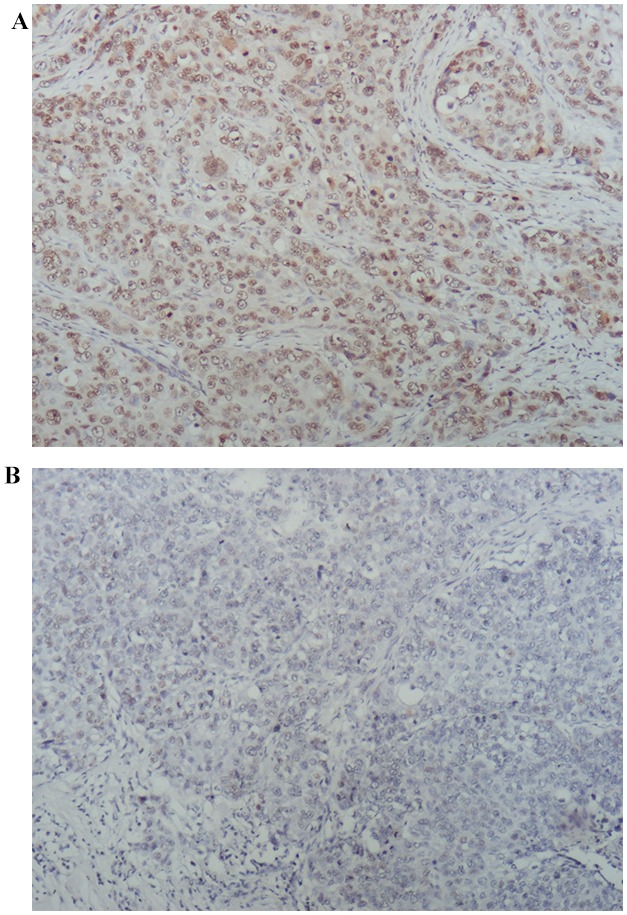
PARP1 expression by IHC staining PARP1expression was evaluated by IHC staining and QS-score: **(A)** high PARP1 expression; and **(B)** low PARP1 expression (×100).

**Figure 4:**
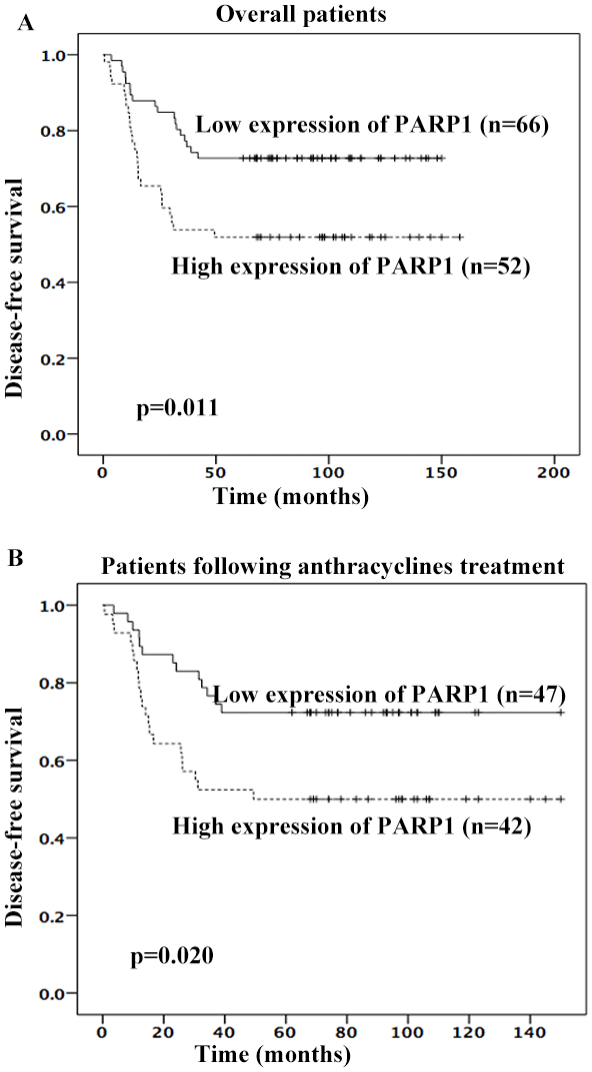
High PARP1 expression was associated with poorer 5-year DFS of TNBC patients High PARP1 expression was associated with poorer 5-year DFS in overall patients **(A)** or patients following anthracyclines treatment **(B)**.

Next, PARP1 expression was determined in the tumors of TNBC patients using immunohistochemistry (IHC) (Figure 3). Because 7 TNBC tissues were not enough for IHC, PARP1 expression was evaluated in 118 patients. The percentage of high PARP1 expression was 44.06%. PARP1 expression was higher in patients with poor prognoses (Table 3). Patients with high PARP1 expression showed poorer 5-year DFS than those with low PARP1 (adjusted HR = 2.236, 95% CI, 1.209 – 4.136, *p* = 0.010; adjusted by menopausal status, Ki67 index, T stage, and lymph node status) (Figure 4A). Patients received adjuvant anthracyclines chemotherapy with high PAPR1 expression (adjusted HR = 2.364, 95% CI, 1.177 – 4.749, *p* = 0.016) exhibited poorer 5-year DFS (Figure 4B).

**Table 3:** PARP1 expression in TNBC patients

Group	Patients with good prognoses	Patients with poor prognoses	P^*^
**PARP1 high expression**	27 (36%)	25 (58.1%)	0.020
**PARP1 low expression**	48 (64%)	18 (41.9%)	

^*^χ^2^ test.

### MiR-221-3p regulated PARP1 expression by directly targeting its 3’-UTR

**Figure 5:**
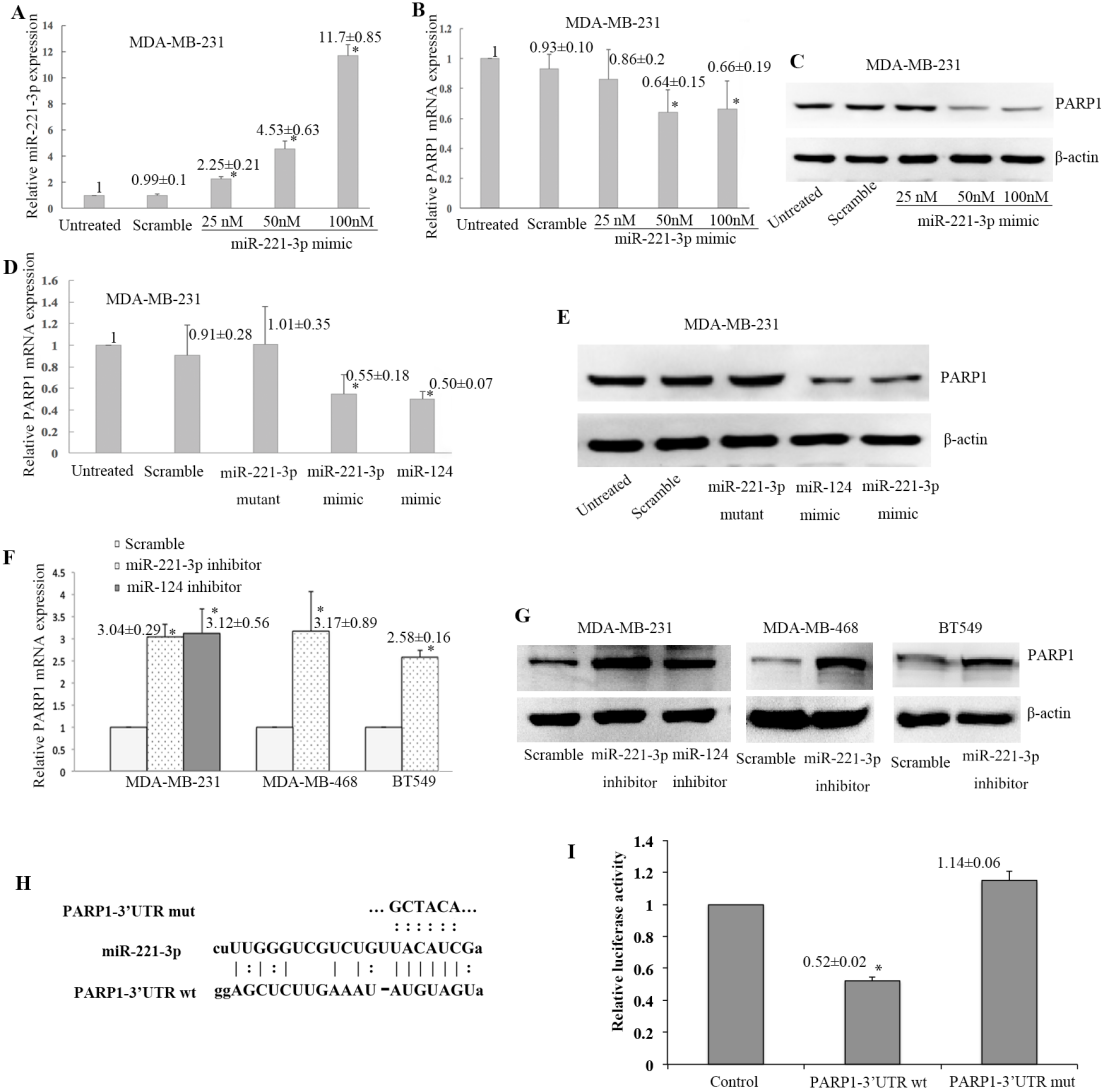
Negative regulation of PARP1 expression by miR-221-3p MiR-221-3p mimic with different doses were transfected into MDA-MB-231 cell line **(A-C)**, and relative miR-221-3pexpression to untreated control was increased in cells with different doses (^*^p < 0.05, t test, A). Relative PARP1 mRNA (^*^p < 0.05, t test, B) and protein (C) expression to untreated control were decreased in transfected dose with 50 nM or 100 nM, but there was no difference between 50 nM transfected cells and 100 nM transfected cells. Then 50 nM of miR-221-3p mimics was chosen to continue the experiments in the MDA-MB-231 cell line **(D-E)**, which showed PARP1 mRNA (^*^p <0.05, t test, D) and protein levels (E) were markedly down-regulated. MiR-124 mimic served as the positive control. Transfection with the miR-221-3p inhibitor increased the PARP1 mRNA (^*^p< 0.05, *t*-test, **F**) and protein **(G)** levels in three TNBC cell lines. **(H)** Sequences of miR-221-3p, PARP1-3’UTR, and mutant PARP1-3’UTR in the dual luciferase experiment. **(I)** Cotransfection with the luciferase constructs containing the wild-type *PARP1* 3’-UTR and miR-221-3p precursor resulted in 48% decline in the luciferase activity compared with the control cells (^*^ p< 0.05).

Next, miR-221-3p and PARP1 expression were determined in TNBC cell lines. First, miR-221-3p mimic with different doses were transfected into MDA-MB-231 cell line. Compared to untreated cells, miR-221-3p expression was increased by 2.25-fold in miR-221-3p mimic transfection cells with 25 nM, 4.53-fold in 50 nM, 11.7-fold in 100 nM (Figure 5A). Relative PARP1 mRNA and protein were decreased in transfected dose with 50 nM or 100 nM, but there was no difference between 50 nM transfected cells and 100 nM transfected cells (Figure 5B, 5C). Therefore, we chose 50 nM of miR-221-3p mimics to evaluate the regulatory role to PARP1. MiR-221-3p mimic or miR-221-3p mutant mimic were transfected into the MDA-MB-231 cell line. MiR-124 was reported to regulate PARP1 expression in breast cancer cells [15]. Thus, we used it as the positive control. Transfection with the miR-221-3p mimic reduced the PARP1 mRNA and protein (Figure 5D, 5E). In contrast, transfection with miR-221-3p inhibitor increased the level of PARP1 mRNA and protein in MDA-MB-231 cell line (Figure 5F, 5G). Similar results were found in MDA-MB-468 and BT549 cell lines.

To determine whether miR-221-3p regulates *PARP1* through the predicted binding sites in its 3’-UTR, we designed two reporter plasmids by incorporating either the wild-type or mutant 3’-UTR of *PARP1* (Figure 5H), which constitutively express luciferase unless repressed by the incorporated 3’-UTR. Cotransfection with the luciferase constructs containing the wild-type *PARP1* 3’-UTR and miR-221-3p precursor resulted in 48% decline in the luciferase activity compared with the control cells (*p* < 0.05, Figure 5I). The above analysis indicates that miR-221-3p negatively regulates PARP1 expression by binding to its 3’-UTR.

## DISCUSSION

Existing studies on miRNAs in TNBC have focused on the difference in miRNA expression profiles between TNBC and non-TNBC cell lines and tissues. In TNBC, multiple miRNAs may be associated with the prognosis of TNBC patients [20]. In the current study, we performed miRNA expression profiling in TNBC tissues of 40 patients with different prognoses in the experimental group. Results revealed that 34 miRNAs were down-regulated in patients with poor prognoses. We selected 5 miRNAs to further verify in the experimental and validation groups by real-time PCR. The expression level of miR-221-3p in cancer tissue was markedly lower in patients with poor prognoses. *PARP1* may be a target gene of miR-221-3p, which down-regulates PARP1 expression possibly by targeting its 3'-UTR.

Dysregulation of miR-221 has been found in various types of cancer, and played different prognostic roles dependent on cancer type. High miR-221 expression shows better prognosis in prostate [21] and ovarian cancers [22], but shows poorer prognosis in gastric [23, 24], liver [25], renal [26], colon [27] cancers and in osteosarcoma [28], glioma [29], melanoma [30] and leukemia [31] (Table 4). In breast cancer, two studies (German and Swede, n=86; Egyptian, n=76) showed poorer prognosis in patients with high miR-221 expression [32, 33]. One study based on TNBC (Greek, n=49) found no correlation between miR-221 expression and prognosis [9]. Another two independent studies based on American (n=473) [34] and Japanese (HER2-negative, n=171) [35] showed that patients with high miR-221 expression had better prognosis, which are consistent with our results of TNBC (n=125). Thus, miRNAs act as oncogenes or tumor suppressors in a context dependent manner, even in the same type of cancer [5, 36]. Several groups reported that miR-221 promoted progression and stemness of breast cancer cell lines [37–41], but results from cell lines may be different from the clinical patients. An oncogene may promote malignant development of cancer cells, but patient prognosis may be improved after clinical treatment. For example, the prognoses of HER2-positive subtype patients after Herceptin treatment were better than TNBC patients [42]. In the present study, through targeting *PARP1*, miR-221 may function as a PARP1 inhibitor, which affected prognosis of TNBC patients.

**Table 4:** The correlation between miR-221 expression and prognosis of cancer patients

Reference	Cases (Characteristics)	Ethnicity	Endogenous control	Prognostic value
Breast cancer				
Eissa, 2015, *Gene*	Breast cancer, n=76; (Luminal A, n=35; Luminal B, n=12; TNBC, n=19; HER2-positive, n=10)	Egyptian	SNORD-68	High miR-221 expression had worse 5-year RFS (HR = 14.84, p = 0.01) [32].
Falkenberg, 2013, *Br J Cancer*	Breast cancer, n=86; (ER+, n=22; HER2+, n=21; LN+, n=38)	German and Swede	RNU43, RNU44	High miR-221 predicted worse prognosis in all (HR=2.57, p=0.028), HER2 positive (p=0.0013) or LN positive (p=0.012) cancers [33].
Radojicic, 2011, *Cell Cycle*	Breast cancer, n=49 (All cases were TNBC.)	Greek	RNU5A, RNU6B	miR-221 expression was not associated with DFS (p=0.4905) or OS (p=0.4578) [9].
Hanna, 2012, *Biotechniques*	Breast cancer, n=473; (ER+, 50%; PR+, 47%; HER2-,77%)	American	U6	High expression of miR-221 predicted better OS (HR=0.702, p=0.031) [34].
Yoshimoto, 2011, *Breast Cancer Res Treat*	Breast, n=171; (HER2-, n=171; ER+, n=132)	Japanese	RNU6B	Patients with high miR-221 exhibited a trend for better OS (HR=0.94, p=0.06) [35].
**Other types of cancers**				
Zheng, 2014, *Prostate*	Prostate cancer, n=118	American	U6	Lower miR-221 was associated with a higher risk of recurrence [21].
Wu, 2017, *Biochem Biophys Res Commun*.	Epithelial ovarian cancer, n=74;	Chinese	U6	High miR-221 had a better OS (HR= 0.3950, p=0.0093) [22].
Smid, 2016, *Int J Oncol.*	Gastric cancer, n=54	Czech	RNU6B	High expression of miR-221 relate to shorter TTP [23].
Liu, 2012, *J Int Med Res.*	Gastric cancer, n=92	Chinese	U6	High miR-221 predicted shorter OS [24].
Li, 2011, *Biochem Biophys Res Commun.*	Hepatocellular carcinoma, n=46	Chinese	mmu-miR-295	High miR-221 was associated with shorter OS [25].
Khella, 2015, *Mol Ther.*	Renal Cell Carcinoma, n=57	Canadian	U6, RNU48, RNU44	High miR-221 was associated with a poor PFS [26].
Cai, 2015, *Int J Clin Exp Med.*	Colon cancer, n=182	Chinese	RNU6B	High miR-221 was associated with a shorter OS [27].
Yang, 2015, *Biomed Pharmacother*	Osteosarcoma, n=108	Chinese	U6	High miR-221 level was correlated with shorter RFS and OS [28].
Zhang, 2016, *Mol Neurobiol.*	Glioma, n=50	Chinese	miR-16	High miR-221 predicted shorter OS [29].
Li, 2014, *Med Sci Monit.*	Melanoma, n=72	Chinese	miR-16	High miR-221 was associated with shorter DFS and OS [30].
Gimenes-Teixeira, 2013, Exp Hematol Oncol.	T-cell acute lymphoblastic leukemia, n=48	Brazilian	RNUs 6B, 19, 38B and 66	High miR-221 predicted shorter OS [31].

DFS, Disease free survival; OS, overall survival; DMFS, Distant metastases free survival.

RFS, Relapse free survival; TTP, time to progression; PFS, progression-free survival.

Because miR-221 and miR-222 are on the same transcriptional units [10], we also detected miR-222-3p expression in paired cancer and normal tissues of 12 TNBC patients from the control group. No correlation between miR-221-3p and miR-222-3p was found in the 12 TNBC cancer tissues. Moreover, the expression level of miR-222-3p in cancer tissues was similar to that in normal tissues, so the prognostic role of miR-222-3p was not evaluated. A larger number of samples are required in order to determine the prognostic role of miR-222-3p expression in breast cancer.

Besides targeting *PARP1*, multiple target genes of miR-221 were validated. MiR-221 inhibited cell cycle by targeting p27^KIP1^ [43] and p57^KIP2^ [44]; and repressed apoptosis by directly targeting pro-apoptotic genes, such as *PUMA* [45] and *BMF* [46]. MiR-221 regulated tumor suppressor genes *PTEN* [19], *TRPS1* [47], *SOCS1* and *CDKN1B* [48], and also directly regulated the expression of two oncogenes, *IRF2* and *SOCS3* [49]. In TNBC cells, miR-221 modulates cell migration and epithelial-mesenchymal transition (EMT) by down-regulating E-cadherin [10].

PARP1 is a member of the poly-ADP ribose transferase family. It acts as a DNA repair protein that can be activated by binding to the single-strand and double-strand break sites of DNA, and it can further complete DNA repairs [14]. PARP1 is highly expressed in one-third of breast cancer patients. High PARP1 expression is an independent prognostic factor for breast cancer recurrence and death [50]. In the current study, the percentage of high PARP1 expression was 44.06% and high PARP1 expression predicted poorer 5-year DFS in TNBC patients. And in patients who received adjuvant anthracyclines chemotherapy, low miR-221-3p or high PARP1 expression was associated with poorer 5-year DFS.

Because PARP1 works in cooperation with BRCA1/2 in homologous recombination repair, homologous deficient cells, such as BRCA1/2 deficient cells, are sensitive to PARP1 inhibitors [16]. Currently, there are more than 50 clinical trials of PARP inhibitors for the treatment of breast, ovarian, prostate, and lung cancers. A phase II clinical trial of olaparib in *BRCA*-mutant advanced breast cancer patients showed that the objective response rates were 41% [51]. Another phase II clinical trial of olaparib monotherapy for *BRCA1/2*- mutant advanced solid tumors showed that the overall complete response and partial response rate was 26.2% [52]. These results demonstrate the potential application value of PARP1 inhibitors, as represented by olaparib, for TNBC. The results obtained in the current study suggest that miR-221-3p down-regulates *PARP1* expression by targeting its 3'-UTR. MiR-221-3p likely plays a similar role as PARP1 inhibitor, and high miR-221-3p expression possibly improves the prognosis of patients by regulating *PARP1* expression.

There were a few limitations in this study. First, this study was a small-sample single-center study. It is necessary to further evaluate the prognostic value of miR-221-3p in TNBC among large-sample, prospective, well-controlled patients with long-term follow-up. Second, because of the sample limitation, RNU6B was the only internal control for the relative expression of miR-221 by RT-PCR. Further study on miR-221 expression should use more internal controls. Third, the regulatory mechanism of miR-221-3p and *PARP1* has not been clearly elucidated. Fourth, in-depth evaluation is needed concerning the mechanism of PARP1 inhibitors and molecular targets of efficacy judgment. Fifth, other miRNAs and related targets should be studied in TNBC patients with different prognoses.

In conclusion, low miR-221-3p and high PARP1 expression levels may contribute to the poor outcome of TNBC patients. MiR-221-3p likely plays a role as a PARP1 inhibitor by directly regulating *PARP1* expression, thereby affecting the prognosis of TNBC patients. This study provides new ideas for more comprehensive studies of prognostic factors and targeted therapy of TNBC. It is expected to provide a theoretical basis for PARP1 inhibitor applications in TNBC and to further improve the treatment effects of TNBC.

## MATERIALS AND METHODS

### Patients and samples

More than 11,000 patients were registered in the Breast Cancer Information Management System of West China Hospital, Sichuan University [53, 54]. We screened 708 cases of patients with TNBC during the period from 2000 to 2012. Patients (a total of 583 cases) who failed follow-up, underwent neoadjuvant chemotherapy, lacked complete clinical information or were unable to provide a sufficient amount of tumor tissue sample were excluded from the study. In total, the study included 125 patients with early TNBC. This study was approved by the Ethics Committee of Clinical Trials and Biomedical Research at West China Hospital. Written informed consent was obtained from each patient. Postoperative therapy was performed according to the standard treatment of National Comprehensive Cancer Network (NCCN) guidelines. The follow-up ended in March 2015. DFS was defined as the interval between surgery date and first relapse of cancer, breast cancer-related death, or last follow-up.

We defined patients with DFS longer than five years as good prognoses and those with recurrence and metastasis as poor prognoses. We randomly selected 20 cases each from the poor prognosis and good prognosis patients to form the experimental group (20 cases with good prognoses vs. 20 cases with poor prognoses). The remaining 85 cases served as the validation group (58 cases with good prognoses vs. 27 cases with poor prognoses). Postoperative paraffin-embedded specimens were obtained from the Department of Pathology, West China Hospital, Sichuan University. Each specimen was serially sectioned into 120μm-thick sections for RNA extraction and subsequent miRNA expression profiling using microarray and real-time PCR analyses. The clinical and pathological information of these 125 patients is summarized in Table 1. In addition to the 125 patients in experimental and validation groups, we also selected 12 TNBC patients with paired cancer and paracancerous normal tissues as the control group.

### Cell culture and treatments

TNBC cell lines (MDA-MB-231, MDA-MB-468, and BT549) were purchased from the Cell Resource Center, Chinese Academy of Medical Sciences. The miR-221-3p mimic, miR-221-3p-mutant and scramble control, miR-221-3p inhibitor and scramble control, miR-124 mimic and inhibitor were purchased from GeneCopoeia (Rockville, MD, USA). Cells were transfected with these oligonucleotides using Lipofectamine 2000 (Invitrogen, Carlsbad, CA, USA) following the manufacturer's instructions.

### MiRNA microarray

Total RNA was extracted from paraffin-embedded tissue specimens using the TRIzol Reagent (Invitrogen, Carlsbad, CA, USA) and an RNeasy Mini Kit (Qiagen, Germantown, MD, USA). RNA samples were labeled and hybridized using a miRCURY^™^ Hy3^™^/Hy5^™^ Power labeling kit (Exiqon, Vedbaek, Denmark) and a miRCURY^™^ LNA Array (v.18.0, Exiqon). The fluorescence intensity of the microarray was scanned using a GenePix 4000B microarray scanner. The original image intensity was determined using GenePix Pro V6.0.

### Real-time PCR

#### MiRNA

RNA was extracted from paraffin-embedded specimens using the miRNeasy FFPE Kit (Qiagen). Subsequently, cDNA synthesis and PCR amplification were performed using the All-in-One^™^ miRNA qRT-PCR Detection Kit (GeneCopoeia). Primers for miR-221-3p (HmiRQP0338), miR-222-3p (HmiRQP0339), miR-34a-3p (HmiRQP0440), miR-203 (HmiRQP0305), miR-4532 (HmiRQP2077), miR-140-5p (HmiRQP0181), and RNU6B (HmiRQP9001) were obtained from GeneCopoeia. RNU6B served as the normalization control. Real-time PCR analyses were carried out using chromo4 (Bio-rad).

#### mRNA

RNA was extracted from the cell line using an RNeasy Mini Kit (Qiagen). Sso Fast Eva Green Supermix was used in the real-time PCR reaction. *PARP1*: upstream primer 5'-CCCTAAAGGCTCAGAACG-3'; downstream primer 5'-CAAGATCGCCGACTCCC-3'. *β-actin*: upstream primer 5'-ACTTAGTTGCGTTACACCCTT-3'; downstream primer 5'-GTCACCTTCACCGTTCCA-3'. Real-time PCR analyses were carried out using chromo4 (Bio-rad).

### Western blotting

The procedure was performed as previously described [54]. Briefly, equal amounts of protein were subjected to 10% polyacrylamide gel electrophoresis and transferred to PVDF membrane. The membrane was incubated with rabbit anti-human PARP1 polyclonal antibody (Cell Signaling Technology, Danvers, MA, USA) or mouse anti-human β-actin monoclonal antibody overnight, followed by goat anti-rabbit or goat anti-mouse secondary antibody (Zsbio, Beijing, China). The bands were visualized using an ECL luminescent reagent (Millipore, Billerica, USA).

### Immunohistochemistry of PARP1 expression

IHC for PARP1(primary rabbit polyclonal antibody against PARP1, ab6079, abcam) was performed using standard procedures. Multiplicative quick score method (QS) was used to assess the expression of PARP1 proteins expression [55]. In brief, the proportion of positive cells was estimated and given a percentage score (1=1–4%; 2=5–19%; 3=20–39%; 4=40–59%; 5=60–79%; and 6=80–100%). The average intensity of the positively staining cells was given an intensity score (0=no staining; 1=weak, 2=intermediate, and 3=strong staining). The QS was calculated by multiplying the percentage score and the intensity score to yield a value from 0 to 18. Based on the QS, PARP1 expression was graded as low (0–9) or high (10–18) (Figure 3).

### Dual luciferase assay

Human embryonic kidney (HEK) 293Ta cells (GeneCopoeia Inc, Rockville, MD, USA) were used for the luciferase assay. HEK-293Ta cells were co-transfected using EndoFectin^™^ Lenti (GeneCopoeia Inc.) with a reporter plasmid containing the wild-type or mutant of *PARP1* inserted downstream of the Gaussian luciferase-secreted reporter gene, the secreted alkaline phosphatase tracking gene (pEZX-MT05, GeneCopoeia Inc), and the pEZX-MR04 plasmid-containing miRNA-221-3p precursor construct or its scrambled control equivalent (GeneCopoeia Inc). The Gaussian luciferase and alkaline phosphatase activities were measured by luminescence in conditioned medium 48 hours after transfection using the secreted-pair dual luminescence kit (GeneCopoeia Inc). The Gaussian luciferase activity was normalized to the alkaline phosphatase activity.

### Statistical analysis

The statistical analysis was performed using the IBM SPSS Statistics software package (version 21.0, IBM-SPSS Statistics, Armonk, NY, USA). Differential expression of miRNAs between TNBC patients with different prognoses was detected using *t* tests or nonparametric tests. The correlation between miRNA expression and clinicopathological factors was evaluated using χ^2^ test. Survival characteristics were performed using the Kaplan-Meier method and log-rank test. The effect of miRNA expression level on prognosis was analyzed by univariate and Cox multivariate risk models. A *t* test was conducted to analyze the differences in target gene mRNA expression levels and HEK293Ta cell fluorescence intensity between different groups. One-way analysis of variance (ANOVA) and Mann-Whitney U tests were run to determine differences in viability between miR-221-3p and scramble transfected cells. A two-sided test P value < 0.05 was judged as statistically significant.

## SUPPLEMENTARY MATERIALS TABLES



## References

[1] Perou CM, Sorlie T, Eisen MB, van de Rijn M, Jeffrey SS, Rees CA, Pollack JR, Ross DT, Johnsen H, Akslen LA, Fluge O, Pergamenschikov A, Williams C (2000). Molecular portraits of human breast tumours. Nature.

[2] Cleator S, Heller W, Coombes RC (2007). Triple-negative breast cancer: therapeutic options. The Lancet Oncology.

[3] Khaled WT, Choon Lee S, Stingl J, Chen X, Raza Ali H, Rueda OM, Hadi F, Wang J, Yu Y, Chin SF, Stratton M, Futreal A, Jenkins NA (2015). BCL11A is a triple-negative breast cancer gene with critical functions in stem and progenitor cells. Nature communications.

[4] Bartel DP (2004). MicroRNAs: genomics, biogenesis, mechanism, and function. Cell.

[5] Lujambio A, Lowe SW (2012). The microcosmos of cancer. Nature.

[6] Gilad S, Lithwick-Yanai G, Barshack I, Benjamin S, Krivitsky I, Edmonston TB, Bibbo M, Thurm C, Horowitz L, Huang Y, Feinmesser M, Hou JS, St Cyr B (2012). Classification of the four main types of lung cancer using a microRNA-based diagnostic assay. The Journal of molecular diagnostics.

[7] Kleivi Sahlberg K, Bottai G, Naume B, Burwinkel B, Calin GA, Borresen-Dale AL, Santarpia L (2015). A serum microRNA signature predicts tumor relapse and survival in triple-negative breast cancer patients. Clinical cancer research.

[8] Miller TE, Ghoshal K, Ramaswamy B, Roy S, Datta J, Shapiro CL, Jacob S, Majumder S (2008). MicroRNA-221/222 confers tamoxifen resistance in breast cancer by targeting p27Kip1. The Journal of biological chemistry.

[9] Radojicic J, Zaravinos A, Vrekoussis T, Kafousi M, Spandidos DA, Stathopoulos EN (2011). MicroRNA expression analysis in triple-negative (ER, PR and Her2/neu) breast cancer. Cell cycle.

[10] Nassirpour R, Mehta PP, Baxi SM, Yin MJ (2013). miR-221 promotes tumorigenesis in human triple negative breast cancer cells. PloS one.

[11] Patnaik SK, Kannisto E, Knudsen S, Yendamuri S (2010). Evaluation of microRNA expression profiles that may predict recurrence of localized stage I non-small cell lung cancer after surgical resection. Cancer research.

[12] Haince JF, Kozlov S, Dawson VL, Dawson TM, Hendzel MJ, Lavin MF, Poirier GG (2007). Ataxia telangiectasia mutated (ATM) signaling network is modulated by a novel poly(ADP-ribose)-dependent pathway in the early response to DNA-damaging agents. The Journal of biological chemistry.

[13] Albert JM, Cao C, Kim KW, Willey CD, Geng L, Xiao D, Wang H, Sandler A, Johnson DH, Colevas AD, Low J, Rothenberg ML, Lu B (2007). Inhibition of poly(ADP-ribose) polymerase enhances cell death and improves tumor growth delay in irradiated lung cancer models. Clinical cancer research.

[14] Heitz F, Harter P, Ewald-Riegler N, Papsdorf M, Kommoss S, du Bois A (2010). Poly(ADP-ribosyl)ation polymerases: mechanism and new target of anticancer therapy. Expert review of anticancer therapy.

[15] Chen SM, Chou WC, Hu LY, Hsiung CN, Chu HW, Huang YL, Hsu HM, Yu JC, Shen CY (2015). The Effect of MicroRNA-124 Overexpression on Anti-Tumor Drug Sensitivity. PloS one.

[16] Streppel MM, Pai S, Campbell NR, Hu C, Yabuuchi S, Canto MI, Wang JS, Montgomery EA, Maitra A (2013). MicroRNA 223 is upregulated in the multistep progression of Barrett's esophagus and modulates sensitivity to chemotherapy by targeting PARP1. Clinical cancer research.

[17] Yang H, Fang F, Chang R, Yang L (2013). MicroRNA-140-5p suppresses tumor growth and metastasis by targeting transforming growth factor beta receptor 1 and fibroblast growth factor 9 in hepatocellular carcinoma. Hepatology.

[18] Bianchini G, Balko JM, Mayer IA, Sanders ME, Gianni L (2016). Triple-negative breast cancer: challenges and opportunities of a heterogeneous disease. Nature reviews Clinical oncology.

[19] Chun-Zhi Z, Lei H, An-Ling Z, Yan-Chao F, Xiao Y, Guang-Xiu W, Zhi-Fan J, Pei-Yu P, Qing-Yu Z, Chun-Sheng K (2010). MicroRNA-221 and microRNA-222 regulate gastric carcinoma cell proliferation and radioresistance by targeting PTEN. BMC cancer.

[20] Yang F, Zhang W, Shen Y, Guan X (2015). Identification of dysregulated microRNAs in triple-negative breast cancer (review). International journal of oncology.

[21] Zheng Q, Peskoe SB, Ribas J, Rafiqi F, Kudrolli T, Meeker AK, De Marzo AM, Platz EA, Lupold SE (2014). Investigation of miR-21, miR-141, and miR-221 expression levels in prostate adenocarcinoma for associated risk of recurrence after radical prostatectomy. The Prostate.

[22] Wu Q, Ren X, Zhang Y, Fu X, Li Y, Peng Y, Xiao Q, Li T, Ouyang C, Hu Y, Zhang Y, Zhou W, Yan W (2017). MiR-221-3p targets ARF4 and inhibits the proliferation and migration of epithelial ovarian cancer cells. Biochemical and biophysical research communications.

[23] Smid D, Kulda V, Srbecka K, Kubackova D, Dolezal J, Daum O, Kucera R, Topolcan O, Treska V, Skalicky T, Pesta M (2016). Tissue microRNAs as predictive markers for gastric cancer patients undergoing palliative chemotherapy. Int J Oncol.

[24] Liu K, Li G, Fan C, Diao Y, Wu B, Li J (2012). Increased Expression of MicroRNA-221 in Gastric Cancer and Its Clinical Significance. The Journal of International Medical Research.

[25] Li J, Wang Y, Yu W, Chen J, Luo J (2011). Expression of serum miR-221 in human hepatocellular carcinoma and its prognostic significance. Biochemical and biophysical research communications.

[26] Khella HW, Butz H, Ding Q, Rotondo F, Evans KR, Kupchak P, Dharsee M, Latif A, Pasic MD, Lianidou E, Bjarnason GA, Yousef GM (2015). miR-221/222 Are Involved in Response to Sunitinib Treatment in Metastatic Renal Cell Carcinoma. Molecular therapy.

[27] Cai K, Shen F, Cui JH, Yu Y, Pan HQ (2015). Expression of miR-221 in colon cancer correlates with prognosis. Int J Clin Exp Med.

[28] Yang Z, Zhang Y, Zhang X, Zhang M, Liu H, Zhang S, Qi B, Sun X (2015). Serum microRNA-221 functions as a potential diagnostic and prognostic marker for patients with osteosarcoma. Biomedicine & pharmacotherapy.

[29] Zhang R, Pang B, Xin T, Guo H, Xing Y, Xu S, Feng B, Liu B, Pang Q (2016). Plasma miR-221/222 Family as Novel Descriptive and Prognostic Biomarkers for Glioma. Molecular neurobiology.

[30] Li P, He QY, Luo CQ, Qian LY (2014). Circulating miR-221 expression level and prognosis of cutaneous malignant melanoma. Medical science monitor.

[31] Gimenes-Teixeira HL, Lucena-Araujo AR, Dos Santos GA, Zanette DL, Scheucher PS, Oliveira LC, Dalmazzo LF, Silva-Júnior WA, Falcão RP, Rego EM (2013). Increased expression of miR-221 is associated with shorter overall survival in T-cell acute lymphoid leukemia. Exp Hematol Oncol.

[32] Eissa S, Matboli M, Sharawy A, El-Sharkawi F (2015). Prognostic and biological significance of microRNA-221 in breast cancer. Gene.

[33] Falkenberg N, Anastasov N, Rappl K, Braselmann H, Auer G, Walch A, Huber M, Hofig I, Schmitt M, Hofler H, Atkinson MJ, Aubele M (2013). MiR-221/-222 differentiate prognostic groups in advanced breast cancers and influence cell invasion. British journal of cancer.

[34] Hanna JA, Wimberly H, Kumar S, Slack F, Agarwal S, Rimm DL (2012). Quantitative analysis of microRNAs in tissue microarrays by *in situ* hybridization. BioTechniques.

[35] Yoshimoto N, Toyama T, Takahashi S, Sugiura H, Endo Y, Iwasa M, Fujii Y, Yamashita H (2011). Distinct expressions of microRNAs that directly target estrogen receptor alpha in human breast cancer. Breast cancer research and treatment.

[36] Chen Q, Si Q, Xiao S, Xie Q, Lin J, Wang C, Chen L, Chen Q, Wang L (2013). Prognostic significance of serum miR-17-5p in lung cancer. Medical oncology.

[37] Howe EN, Cochrane DR, Richer JK (2012). The miR-200 and miR-221/222 microRNA families: opposing effects on epithelial identity. Journal of mammary gland biology and neoplasia.

[38] Falkenberg N, Anastasov N, Schaub A, Radulovic V, Schmitt M, Magdolen V, Aubele M (2015). Secreted uPAR isoform 2 (uPAR7b) is a novel direct target of miR-221. Oncotarget.

[39] Roscigno G, Quintavalle C, Donnarumma E, Puoti I, Diaz-Lagares A, Iaboni M, Fiore F, Russo V, Todaro M, Romano G, Thomas R, Cortino G, Gaggianesi M (2016). MiR-221 promotes stemness of breast cancer cells by targeting DNMT3b. Oncotarget.

[40] Li B, Lu Y, Wang H, Han X, Mao J, Li J, Yu L, Wang B, Fan S, Yu X, Song B (2016). miR-221/222 enhance the tumorigenicity of human breast cancer stem cells via modulation of PTEN/Akt pathway. Biomedicine & Pharmacotherapy.

[41] Pan Y, Li J, Zhang Y, Wang N, Liang H, Liu Y, Zhang CY, Zen K, Gu H (2016). Slug-upregulated miR-221 promotes breast cancer progression through suppressing E-cadherin expression. Scientific reports.

[42] Hennigs A, Riedel F, Gondos A, Sinn P, Schirmacher P, Marme F, Jager D, Kauczor HU, Stieber A, Lindel K, Debus J, Golatta M, Schutz F (2016). Prognosis of breast cancer molecular subtypes in routine clinical care: A large prospective cohort study. BMC cancer.

[43] Galardi S, Mercatelli N, Giorda E, Massalini S, Frajese GV, Ciafre SA, Farace MG (2007). miR-221 and miR-222 expression affects the proliferation potential of human prostate carcinoma cell lines by targeting p27Kip1. The Journal of biological chemistry.

[44] Medina R, Zaidi SK, Liu CG, Stein JL, van Wijnen AJ, Croce CM, Stein GS (2008). MicroRNAs 221 and 222 bypass quiescence and compromise cell survival. Cancer research.

[45] Zhang J, Han L, Ge Y, Zhou X, Zhang A, Zhang C, Zhong Y, You Y, Pu P, Kang C (2010). miR-221/222 promote malignant progression of glioma through activation of the Akt pathway. International journal of oncology.

[46] Gramantieri L, Fornari F, Ferracin M, Veronese A, Sabbioni S, Calin GA, Grazi GL, Croce CM, Bolondi L, Negrini M (2009). MicroRNA-221 targets Bmf in hepatocellular carcinoma and correlates with tumor multifocality. Clinical cancer research.

[47] Stinson S, Lackner MR, Adai AT, Yu N, Kim HJ, O'Brien C, Spoerke J, Jhunjhunwala S, Boyd Z, Januario T, Newman RJ, Yue P, Bourgon R (2011). TRPS1 targeting by miR-221/222 promotes the epithelial-to-mesenchymal transition in breast cancer. Science signaling.

[48] Li Y, Liang C, Ma H, Zhao Q, Lu Y, Xiang Z, Li L, Qin J, Chen Y, Cho WC, Pestell RG, Liang L, Yu Z (2014). miR-221/222 promotes S-phase entry and cellular migration in control of basal-like breast cancer. Molecules.

[49] Kneitz B, Krebs M, Kalogirou C, Schubert M, Joniau S, van Poppel H, Lerut E, Kneitz S, Scholz CJ, Strobel P, Gessler M, Riedmiller H, Spahn M (2014). Survival in patients with high-risk prostate cancer is predicted by miR-221, which regulates proliferation, apoptosis, and invasion of prostate cancer cells by inhibiting IRF2 and SOCS3. Cancer research.

[50] Rojo F, Garcia-Parra J, Zazo S, Tusquets I, Ferrer-Lozano J, Menendez S, Eroles P, Chamizo C, Servitja S, Ramirez-Merino N, Lobo F, Bellosillo B, Corominas JM (2012). Nuclear PARP-1 protein overexpression is associated with poor overall survival in early breast cancer. Annals of oncology.

[51] Tutt A, Robson M, Garber JE, Domchek SM, Audeh MW, Weitzel JN, Friedlander M, Arun B, Loman N, Schmutzler RK, Wardley A, Mitchell G, Earl H (2010). Oral poly(ADP-ribose) polymerase inhibitor olaparib in patients with BRCA1 or BRCA2 mutations and advanced breast cancer: a proof-of-concept trial. Lancet.

[52] Kaufman B, Shapira-Frommer R, Schmutzler RK, Audeh MW, Friedlander M, Balmana J, Mitchell G, Fried G, Stemmer SM, Hubert A, Rosengarten O, Steiner M, Loman N (2015). Olaparib monotherapy in patients with advanced cancer and a germline BRCA1/2 mutation. Journal of clinical oncology.

[53] Deng L, Chen J, Zhong XR, Luo T, Wang YP, Huang HF, Yin LJ, Qiu Y, Bu H, Lv Q, Zheng H (2015). Correlation between activation of PI3K/AKT/mTOR pathway and prognosis of breast cancer in Chinese women. PloS one.

[54] Zhong X, Xie G, Zhang Z, Wang Z, Wang Y, Wang Y, Qiu Y, Li L, Bu H, Li J, Zheng H (2016). MiR-4653-3p and its target gene FRS2 are prognostic biomarkers for hormone receptor positive breast cancer patients receiving tamoxifen as adjuvant endocrine therapy. Oncotarget.

[55] Zhai L, Li S, Li X, Li H, Gu F, Guo X, Liu F, Zhang X, Fu L (2015). The nuclear expression of poly (ADP-ribose) polymerase-1 (PARP1) in invasive primary breast tumors is associated with chemotherapy sensitivity. Pathology, research and practice.

