# Supplementary material for: Downregulation of miR-221-3p and upregulation of its target gene PARP1 are prognostic biomarkers for triple negative breast cancer patients and associated with poor prognosis
Source: Oncotarget. 2017 Oct 6;8(65):108712–25. doi: 10.18632/oncotarget.21561 (PMC5752475; doi:10.18632/oncotarget.21561)
Supplement: Supplementary file 1 [file oncotarget-08-108712-s001.pdf]

Downregulation of miR-221-3p and upregulation of its target gene PARP1 are prognostic biomarkers for triple negative breast cancer patients and associated with poor prognosis

SUPPLEMENTARY MATERIALS

Supplementary Table 1: List of miRNAs showing significant differential expression in cancer tissue of patients with different prognoses

Downregulated miRNAs in poor-prognosis patients	Difference in fold	*P*
hsa-miR-9-5p	0.145346	0.01362963
hsa-miR-34a-3p	0.161597	0.01458107
hsa-miR-203	0.190439	0.0043215
hsa-miR-4485	0.203726	0.01926316
hsa-miR-631	0.283303	0.04549965
hsa-miR-221-3p	**0.292138**	***0.01035038***
hsa-miR-4797-5p	0.300028	0.0003767
hsa-miR-4633-5p	0.391095	0.00060561
hsa-miR-4532	0.401721	2.9928E-10
hsa-miR-5189	0.40702	0.00012348
hsa-miR-4524b-5p	0.423808	2.6842E-05
hsa-miR-552	0.429549	0.00070735
hsa-miR-101-3p	0.437309	0.0032341
hsa-miR-1260a	0.438091	0.00028757
hsa-miR-3656	0.445356	0.00013904
hsa-miR-339-5p	0.448556	0.0022623
hsa-miR-302a-3p	0.452909	1.6444E-05
hsa-miR-140-5p	0.501799	0.04555784
hsa-miR-200c-3p	0.502102	0.04816397
hsa-miR-425-5p	0.510293	0.00831287
hsa-miR-1260b	0.514544	0.00028032
hsa-miR-491-3p	0.516834	0.00074857
hsa-miR-21-5p	0.532868	0.00628982
hsa-miR-520f	0.54796	0.03732391
hsa-miR-3913-3p	0.549198	0.05645647
hsa-miR-22-3p	0.559257	0.00861976
hsa-miR-711	0.6037	0.01130327
hsa-miR-1280	0.606175	0.00012181
hsa-miR-26a-5p	0.623142	0.03036584
hsa-miR-3606	0.640556	0.00166496
hsa-miR-2681-5p	0.642853	0.00804246
hsa-miR-4482-3p	0.648905	0.00545933
hsa-miR-3676-5p	0.662995	5.8521E-05
hsa-miR-4500	0.665215	0.025114

Supplementary Table 2: Correlation between miR-221-3p expression and clinicopathological factors

Factor	MiR-221-3p expression		*p*
	Low expression (62 cases)	High expression (63 cases)	
Menopausal status			
Premenopausal	27 (43.5%)	33 (52.4%)	0.323
Postmenopausal	35 (56.5%)	30 (47.6%)	
T stage			
T1/2	54 (87.1%)	53 (84.1%)	0.636
T3/4	8 (12.9%)	10 (15.9%)	
Lymph node metastasis			
No	25 (40.3%)	30 (47.6%)	0.411
Yes	37 (59.7%)	33 (52.4%)	
Ki-67			
<14%	13 (21.0%)	7 (11.1%)	0.133
≥14%	49 (79.0%)	56 (88.9%)	
